# Comparison of effectiveness of Halo-femoral traction after anterior spinal release in severe idiopathic and congenital scoliosis: a retrospective study

**DOI:** 10.1186/1749-799X-2-23

**Published:** 2007-11-30

**Authors:** Yong Qiu, Zhen Liu, Feng Zhu, Bin Wang, Yang Yu, Zezhang Zhu, Bangping Qian, Weiwei Ma

**Affiliations:** 1 Spine Surgery, Drum Tower Hospital, Nanjing University Medical School, Nanjing, China

## Abstract

**Background:**

Halo-femoral traction could gradually improve the coronal and sagittal deformity and restore the trunk balance through the elongation of the spine. The purpose of this retrospective study was to assess the effectiveness of Halo-femoral traction after anterior spinal release in the management of severe idiopathic and congenital scoliosis.

**Methods:**

Sixty patients with severe and rigid curve treated with anterior spinal release, Halo-femoral traction, and second stage posterior spinal fusion were recruited for this retrospective study. Idiopathic Scoliosis (IS) group was 30 patients (23 females and 7 males) with mean age of 15.5 years. The average coronal Cobb angle was 91.6° and the mean global thoracic kyphosis was 50.6°. The curve type of these patients were 2 with Lenke 1AN, 4 with Lenke 1A+, 1 with Lenke 1BN, 10 with Lenke 1CN, 3 with Lenke 1C+, 3 with Lenke 3CN, 3 with Lenke 3C+, and 4 with Lenke 5C+. Congenital Scoliosis (CS) group included 30 patients (20 females and 10 males) with average age of 15.2 years. The average coronal Cobb angle of the main curve before operation was 95.7° and the average thoracic kyphosis was 70.2°. All patients had a minimum 12-month follow-up radiograph (range 12–72 months, mean 38 months).

**Results:**

The average traction time was 23 days and the average traction weight was 16 kg. Four patients experienced brachial plexus palsy and complete nerve functional restoration was achieved at two months follow-up. For the IS group, the post-operative mean Cobb angle of major curve averaged 40.1° with correction rate of 57.5%. For the CS group, the post-operative mean Cobb angle was 56.5° with average correction rate of 45.2%. The difference in curve magnitude between the IS and CS patients after posterior correction was statistically significant (t = 4.15, p < 0.001). The correction rate of kyphosis between IS and CS patients was also statistically significant (t = -2.59, p < 0.016).

**Conclusion:**

Halo-femoral traction was a safe, well-tolerated and effective method for the treatment of severe and rigid scoliosis patients. The posterior correction rate obtained after anterior release and traction was significant superior than that recorded from side bending film in current study.

## Background

With the usage of third-generation spinal instrumentation such as CDH, ISOLA and TSRH, the curve correction obtained from posterior spinal fusion had a significant improvement [1,2]. However, the management of severe and rigid scoliosis remained a big challenge to spine surgeon. Preoperative traction could be one option to provide better correction of the rigid spinal deformity and minimize neurological complications associated with forceful intra-operative distraction. Some authors had studied the usage of Halo-femoral traction as one of the preparative treatment prior to posterior reconstructive surgery for severe scoliosis, especially for those with respiratory dysfunction. Halo-femoral traction could gradually improve the coronal and sagittal deformity and restore the trunk balance through the elongation of the spine. Respiratory function improvement was also reported [3-5]. The purpose of this retrospective study was to assess the effectiveness of Halo-femoral traction after anterior spinal release in the management of severe idiopathic and congenital scoliosis.

## Methods

A total of 60 patients with severe and rigid curve and with detailed follow-up data were recruited for this retrospective study. All these patients were treated with anterior spinal release, halo-femoral traction and second stage posterior spinal fusion in authors' hospital from August 1998 to May 2005. The inclusive criteria were as following: congenital scoliosis or idiopathic scoliosis; halo-femoral traction only performed after one stage anterior spinal release and removed before posterior surgery; no history of previous spinal surgery and a minimum postoperative follow-up of 12-month. Standing long-cassette antero-posterior (AP) and lateral radiographs of the whole spine were taken before anterior surgery, 10 days, 12-month after posterior surgery and at final follow-up respectively. Coronal Cobb angles were measured on standing AP film and side bending film. Thoracic kyphosis was measured on the lateral radiograph between the upper endplate of T5 vertebra and the lower endplate of T12 vertebra using the Cobb method [6]. All patients had a minimum 12-month follow-up (range 12–72 months, mean 38 months).

Idiopathic Scoliosis (IS) group included 30 patients (23 females and 7 males). The age at surgery ranged from 10 years to 20 years old with mean age of 15.5 years old. The average coronal Cobb angle was 91.6° (ranged 70°–146°), and the mean global thoracic kyphosis was 50.6° (ranged 26–100°). The curve type of these patients were analyzed [7], and there were 2 with Lenke 1AN, 4 with Lenke 1A+, 1 with Lenke 1BN, 10 with Lenke 1CN, 3 with Lenke 1C+, 3 with Lenke 3CN, 3 with Lenke 3C+, and 4 with Lenke 5C+. Congenital Scoliosis (CS) group had 30 patients (20 females and 10 males). According to the classification of congenital scoliosis [8], 8 patients were classified as defect of formation, 6 patients as defect of segmentation and 16 patients had combined anomaly. The average age of the patients was 15.2 years (ranged 10–20). The average coronal Cobb angle of the main curve was 95.7° (range 70°–150°) and the average thoracic kyphosis was 70.2° (range 28°–155°) pre-operatively.

All sixty patients received first stage anterior spinal release with the traditional thoracotomy approach and post-operative Halo-femoral traction. None of congenital scoliosis patients were experienced excision of hemivertebra. Traction was usually started the second after anterior surgery with a weight of 2 kg and gradually increased at a rate of 2 to 3 pounds per day if patients well tolerated. The maximum traction weight could be 33% to 50% of the whole body weight depending on patients' tolerance. Traction was applied for a minimum of 12 hours per day, with the traction weight lessened to 50% in the night. During the traction, the patient's neurological status was frequently checked. If hyper reflex of the extremities, Babinski sign, paresthesia, dysfunction of cranial nerves or any other neurological compromise were noted, the weight would be immediately reduced. The length of the traction period was mainly determined by the radiographic evidence of curve improvement on weekly radiographs, in addition to clinical evaluation of the patients' pulmonary and neurological function. Second stage posterior corrective surgery with CD, CDH or TSRH instrumentation were performed after Halo-femoral traction were removed and all the sixty patients with scoliosis surgically were treated by hybrid constructs with hooks and screws.

Statistical analysis was performed for each dependent variable comparing the IS *versus *CS patients by an independent group's t test. All tests results with P < 0.05 were considered statistically significant.

## Results

The average days with halo-femoral traction were similar for IS (22 ± 6.3) and CS (25 ± 9.4) patients. The average traction weight was 16 kg, which accounts for 38% (range 15–50%) of patients' total body weight. Four patients suffered from brachial plexus palsy (1 CS patient and 3 IS patients), complete nerve function restoration were achieved at two months follow-up after rehabilitation training.

**Figure 1:**
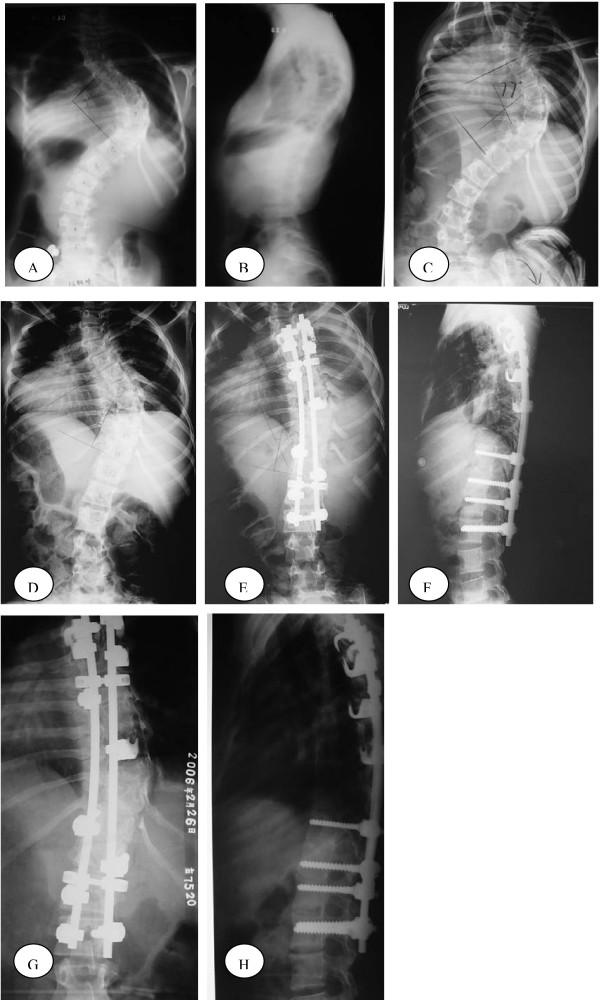
A 14-year-old girl with idiopathic scoliosis and the Lenke classification was 1C+. A-B: AP radiographs before treatment showing right thoracic scoliosis measured 92°. C:Bending films showed right thoracic scoliosis corrected to 77°. D:The right thoracic curve correction obtained with Halo-femoral traction treatment was 40.2%. E-F:The major curve measured 35° after posterior spinal fusion and the correction rate was 62%. G-H: AP and lateral radiographs at 20-month follow-up showed solid spinal fusion with a 37° right thoracic curve.

No significant differences were found between the two groups with respect to age or gender distribution. For the IS group, the average pre-operative major curve magnitude was 91.6°(ranged 70°–146°) and decreased to 71.7°(ranged 45°–120°) on side bending (average correction rate: 24.3%). The major curve averaged 58.1°(range 37°–90°) at the end of the Halo-femoral traction treatment and the average correction rate obtained was 39.3% (ranged 28.6%–50.6%), then improved to 40.1°(ranged 20°–65°) after posterior corrective surgery. The mean Cobb's angle at final follow-up was 42.9° (ranged 24°–66°). The mean loss of correction was 2.9% ± 2.3% (Figure 1).

**Figure 2:**
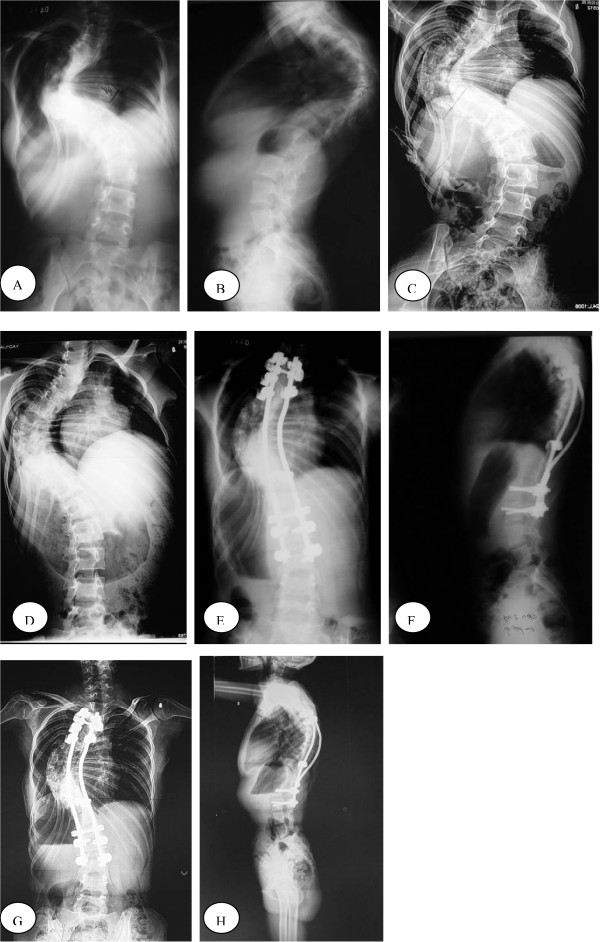
A 13-year-old girl with congenital scoliosis with defect of formation and segmentation. A-B: AP radiographs before treatment showed left thoracic scoliosis measured 98° and thoracic kyphosis measured 85°. C:Bending films showed right thoracic scoliosis only corrected to 90°. D:The left thoracic curve correction obtained with Halo-femoral traction was 24.1%. E-F:The major curve measured 50°after posterior spinal fusion with correction rate of 49.0%. The correction rate of kyphosis was 47.1%. G-H: AP and lateral radiographs at 18-month follow-up showed solid spinal fusion with a 53°left thoracic curve and a 45° thoracic kyphosis.

For the 30 cases with CS, initial coronal Cobb angle averaged 95.7° (range 70°–150°). The curve magnitude on bending film averaged 73.8° (range 45°–130°) with average correction rate of 22.5% (ranged 6.5%–37.8%). At the end of the Halo-femoral traction treatment the Cobb's angle averaged 68.4°(ranged 40°–115°) and the correction rate averaged 35.3% (ranged 23.3%–50.0%). The curve reduced to 56.5° (ranged 35°–110°) immediately after posterior surgery and to 58.9° (ranged 37°–112°) at final follow-up. The average loss of correction was 3.2% ± 2.1%. The average pre-operative coronal Cobb angle and the average time in Halo-femoral traction were similar for IS and CS patients. The difference in curve magnitude between the IS and CS patients after posterior correction was statistically significant (t = 4.15, p < 0.001) (Figure 2).

Improvement on global sagittal alignment was also observed in IS and CS patients. For IS patients, the mean preoperative thoracic kyphosis was 50.6° (ranged 26°–100°), which improved to 30.6° (ranged 22°–50°) after posterior surgery, and was maintained as 31.6° (ranged 21°–52°) at final follow-up. For CS cases, initial mean thoracic kyphosis was 70.2° (ranged 28°–155°) and decreased to 39.0° (ranged 11°–82°) post-operatively. Before anterior release, the magnitude of thoracic kyphosis of CS patients was larger than that of IS cases (t = -2.21, p = 0.041, Table 1). After posterior surgery, the difference of the correction rate of kyphosis between IS and CS patients was also statistically significant (t = -2.59, p < 0.016, Table 1).

**Table 1:** Clinical datas of the IS and CS groups

Group	n	Time in Halo-femoral traction(days)	Initial Coronal Cobb Angle (°)	Thoracic Kyphosis (T5–T12,°)	Curve Correction With Bending film(%)	Cobb Correction After Halo-Femoral Traction (%)	Cobb Correction After Posterior Fusion(%)	Thoracic Kyphosis correction After Posterior Fusion (T5–T12, %)	Loss of Correction (Coronal%)	Loss of Correction (Sagittal%)
IS	30	22 ± 6.3	91.6 ± 20.1	50.6 ± 18.4	24.3 ± 8.2	39.3 ± 6.24	57.5 ± 8.37	33.7 ± 12.8	2.9 ± 2.3	2.3 ± 2.1
CS	30	25 ± 9.4	95.7 ± 24.5	70.2 ± 34.3	22.5 ± 11.7	35.3 ± 7.27	45.2 ± 8.97	43.5 ± 14.2	3.2 ± 2.1	2.5 ± 1.9
t	-	-0.25	-0.27	-2.21	1.34	1.36	4.15	-2.59	-0.73	-1.38
p	-	0.64	0.81	0.041*	0.19	0.18	<0.001*	0.016*	0.28	0.18

*:p < 0.05

## Discussion

With the development of the spinal corrective techniques and the advancement of the instrumentation, severe and rigid scoliosis which used to be difficult to correct became manageable. At present, the definition of severe scoliosis remains controversial. Greiner *et al. *[9]determined that AIS patients did not exhibit clinically significant respiratory symptoms until their curves were 60 to 100°, so he defined severe scoliosis as Cobb angle larger than 60°. Lenke *et al. *[10] have defined it as Cobb angle ≥ 70°, and Tokunaga [11] thought that Cobb angle > 80° could be treated as severe scoliosis. As for the rigid scoliosis, its definition was also unclear until recently. According to author's clinical experience, the results of one stage posterior surgery for the scoliosis with a coronal Cobb angle less than 70 degrees and a flexible index on Bending films more than 40% was satisfactory. Therefore, patients with severe and rigid scoliosis were recruited in current study with a coronal Cobb angle larger than 70° and flexible index on bending films less than 40%.

The aim of the anterior spinal release was to increase spinal flexibility and to improve subsequent correction rate at posterior instrumentation [12,13]. Tokunaga *et al. *[11] reported that staged surgery including anterior release was an effective surgical method for patients with severe scoliosis, where a rigid curve or the risk of neurological complications due to acute forceful correction may exist. Mehlman *et al.*[14]also reported that the spinal release and halo-femoral traction protocol outlined offer a safe, controlled approach to the reduction of severe spine deformities before fusion. In current study all the patients received anterior spinal release first.

Traction as a method of correction of spinal deformity could be dated back to 3500 BC [15]. Perry and Nickel first introduced the halo device in 1959 [16] during which time a jacket or cast was used for caudal support. Then several other count-traction methods were invented: halo-gravity, halo-pelvic and halo-femoral traction [17-19]. In terms of halo-femoral traction, Kane *et al. *[20] reported their series of 30 scoliotic patients in 1967. The average original curve measured 112° and reduced to 58° after final correction. Four patients got pin-site irritation and the pins were reinserted. Paresthesia developed in 3 patients, and 1 had abducens nerve palsy; all the symptoms recovered with the reduction of traction forces. Details about the types of curves treated and specific treatment regimens were not provided in this paper. Bonnett *et al. *[21] reported that preoperative halo-femoral traction resulted in 57% correction of scoliosis as well as 53% correction of pelvic obliquity in 37 patients with paralytic scoliosis. Arlet *et al. *[22] reported on the usage of halo-femoral traction to treat a 17-year-old girl with congenital scoliosis of 145° and cor pulmonale. Correction of the deformity and improvement in pulmonary function were well achieved. Huang *et al. *[15] reported on the usage of intra-operative halo-femoral traction to treat severe scoliosis and associated pelvic obliquity in a 14-year-old patient with cerebral palsy. The patient underwent one stage anterior and posterior spinal fusion, the posterior procedure was performed with the patient under halo-femoral traction. The patient responded well to the surgical intervention and had a stable correction of his pelvic obliquity. Mehlman *et al. *[14] assessed the effectiveness of spinal release and halo-femoral traction in the management of severe spinal deformity in 2004. Twenty-four patients were treated with halo-femoral traction at the interval between anterior spinal release and posterior surgery. The etiology of the deformity included IS, CS, Scheuermann's kyphosis, Neuromuscular scoliosis, and Osteogenesis imperfecta. The correction obtained after Halo-feromal traction averaged 59% (ranged 14–100%).

In current series, compared with CS with similar curve magnitude, the patients with severe and rigid idiopathic scoliosis were slightly more flexible on side bending film (IS 24.3% correction *vs. *CS 22.5%). Curve correction obtained after traction has a significant improvement when compared with the correction obtained from side bending film in our study. This statistically significant difference confirms the efficacy of the technique of Halo-femaral traction. We also found that the average correction obtained from posterior fusion was 57.5% in IS group, significantly higher than that in CS group (45.2%, p < 0.001). Current results demonstrated less overall curve correction rate when compared with the reports of Kane *et al. *[20], Bonnett *et al. *[21] and Mehlman *et al. *[14]. This may be due in part to lower traction forces used in our study (only 36% of the average body weight) than Mehlman study (54% of the average body weight). Furthermore, the curves in Kane and Bonnett's study were less rigid than current study.

Leatherman [23] first described a two-stage procedure for the treatment of congenital scoliosis. In his study, the mean curve correction obtained after the second stage was 45.6% and the correction of kyphosis was 44.4%. Author's results demonstrate that after posterior surgery the curve correction obtained averaged 45.2% and the thoracic kyphosis magnitude decreased to 39.0° (ranged 11°–82°) with average correction rate of 43.5%. Although the curve correction rate in two studies were similar, the initial curve angel of CS patients in current study were far more serious than that in Leatherman's study. Therefore we could conclude that Halo-femaral traction had a enormous effectiveness for the correction of patients with severe and rigid congenital scoliosis.

Severe coronal curve usually associated with significant deformity on sagittal plane. In current study, twenty-three patients with IS and twenty-five CS patients had pre-operative thoracic kyphosis (T5–T12 > 40°). Compared with the IS patients, the mean pre-operative thoracic kyphosis for CS was significant higher (70.2° *vs. *50.6°). Combined with Halo-femoral traction, modern spinal instrumentation system provided good correction on sagittal plane for severe scoliosis. Thoracic kyphosis of patients in our study corrected well after posterior surgery, especially for CS patients.

Complications related to the halo itself included pin loosening, superficial, and deep pin tract infections. Brain abscess has also been previously described with halo pins [24]. Halo-femoral traction compiled certain neurological complications [25]. Rinella [26] reported a total of 42 consecutive patients with severe operative scoliosis, kyphoscoliosis, or kyphosis treated with halo-gravity traction. Triceps palsy (2.38%), and brachial plexus palsy (2.38%) occurred during halo traction. Traction-related complications were also encountered in our study. In the present study, 4 cases suffered from brachial plexus palsy (1 CS patients, 3 IS patients). All patients restored their complete neural function at two months follow-up. The most likely cause of the injury was thought to be due to the hyper-abduction of the arm and over-stretched of the brachial plexus. Brachial plexus palsy associated with Halo-femoral traction in severe and rigid scoliosis was a temporary, revertible damage to nerve function. If the symptoms were promptly detected and rehabilitation training and appropriate medication were prescribed timely, complete nerve functional restoration could be achieved.

Spinal cord injury and paralysis were the most serious complications of spinal corrective surgeries. Cotrel [27] reported that the incidence was 0.8%. Patients with severe and rigid scoliosis were thought to be at greater risk of these complications. Some authors advocated rapid correction *via *one stage anterior release and posterior surgery for patients with severe scoliosis without an intervening period of traction [28]. Long term follow-up and big sample size were mandatory to support these one-stage or "rapid correction" conception. Our results showed that Halo-femoral traction was a safe, well-tolerated and efficacious method in the treatment of this formidable disease. Combined with anterior spinal release and posterior fusion, it could notably reduce the incidence of severe complication such as spinal cord injury, at the time of good correction of severe spinal deformity. In addition, curve correction obtained after traction was significantly superior than that achieved on side bending film in current study, therefore the pre-operative side bending radiography may not able to accurately predict the correction rate of posterior instrumentation for severe scoliosis.

## Conclusion

Halo-femoral traction was a safe and effective method for the treatment of severe idiopathic and congenital scoliosis patients, especially for IS patients. The posterior correction rate obtained after anterior release and traction was significant superior than that recorded from side bending film in current study.

## Competing interests

The author(s) declare that they have no competing interests.
